# Bioinformatics analysis of the expression of potential common genes and immune-related genes between atrial fibrillation and chronic kidney disease

**DOI:** 10.3389/fcvm.2025.1521722

**Published:** 2025-02-26

**Authors:** Jieying Teng, Guoxiong Deng

**Affiliations:** ^1^ Department of Cardiology, The Fifth Affiliated Hospital of Guangxi Medical University, Nanning, China; ^2^ Department of Cardiology, The First People’s Hospital of Nanning, Nanning, China

**Keywords:** atrial fibrillation, chronic kidney disease, bioinformatics, inflammatory, immune process, CXCLs/CXCR

## Abstract

**Research objective:**

This study is based on bioinformatics analysis to explore the co-expressed differentially expressed genes (DEGs) between atrial fibrillation (AF) and chronic kidney disease (CKD), identify the biomarkers for the occurrence and development of the two diseases, investigate the potential connections between AF and CKD, and explore the associations with immune cells.

**Methods:**

We downloaded Two AF gene chip datasets (GSE79768, GSE14975) and two CKD gene chip datasets (GSE37171, GSE120683) from the GEO database. After pre-processing and standardizing the datasets, two DEGs datasets were obtained. The DEGs were screened using R language, and the intersection was taken through Venn diagrams to obtain the co-expressed DEGs of AF and CKD. To obtain the signal pathways where the co-expressed DEGs were significantly enriched, GO/KEGG enrichment analyses were used to analysis the co-expressed DEGs. The Cytoscape software was used to further construct a PPI network and screen key characteristic genes, and the top 15 co-expressed DEGs were screened through the topological algorithm MCC. To further screen key characteristic genes, two machine-learning algorithms, LASSO regression and RF algorithm, were performed to screen key characteristic genes for the two disease datasets respectively to determine the diagnostic values of the characteristic genes in the two diseases. The GeneMANIA online database and Networkanalyst platform were used to construct gene-gene and TFs-gene interaction network diagrams respectively to predict gene functions and find key transcription factors. Finally, the correlation between key genes and immune cell subtypes was performed by Spearman analysis.

**Research results:**

A total of 425 DEGs were screened out from the AF dataset, and 4,128 DEGs were screened out from the CKD dataset. After taking the intersection of the two, 82 co-expressed DEGs were obtained. The results of GO enrichment analysis of DEGs showed that the genes were mainly enriched in biological processes such as secretory granule lumen, blood microparticles, complement binding, and antigen binding. KEGG functional enrichment analysis indicated that the genes were mainly enriched in pathways such as the complement coagulation cascade, systemic lupus erythematosus, and Staphylococcus aureus infection. The top 15 DEGs were obtained through the MCC topological algorithm of Cytoscape software. Subsequently, based on LASSO regression and RF algorithm, the key characteristic genes of the 15 co-expressed DEGs of AF and CKD were further screened, and by taking the intersection through Venn diagrams, five key characteristic genes were finally obtained: PPBP, CXCL1, LRRK2, RGS18, RSAD2. ROC curves were constructed to calculate the area under the curve to verify the diagnostic efficacy of the key characteristic genes for diseases. The results showed that RSAD2 had the highest diagnostic value for AF, and the diagnostic values of PPBP, CXCL1, and RSAD2 for CKD were all at a relatively strong verification level. Based on AUC >0.7, co-expressed key genes with strong diagnostic efficacy were obtained: PPBP, CXCL1, RSAD2. The results of the GeneMANIA online database showed that the two biomarkers, BBPB and CXCL1, mainly had functional interactions with cytokine activity, chemokine receptor activity, cell response to chemokines, neutrophil migration, response to chemokines, granulocyte chemotaxis, and granulocyte migration. The TFs-gene regulatory network identified FOXC1, FOXL1, and GATA2 as the main transcription factors of the key characteristic genes. Finally, through immune infiltration analysis, the results indicated that there were various immune cell infiltrations in the development processes of AF and CKD.

**Research conclusion:**

PPBP, CXCL1, and RSAD2 are key genes closely related to the occurrence and development processes between AF and CKD. Among them, the CXCLs/CXCR signaling pathway play a crucial role in the development processes of the two diseases likely. In addition, FOXC1, FOXL1, and GATA2 may be potential therapeutic targets for AF combined with CKD, and the development of the diseases is closely related to immune cell infiltration.

## 1 Research background

Atrial fibrillation (AF) is the most common arrhythmia in clinical practice. In the past two decades, with the increase in exposure to risk factors, the aging of the population, and the prolonged survival period of patients with chronic diseases (1), AF has brought a heavy disease burden to society and medical services. AF not only affects the quality of life of patients but also increases the risk of complications such as heart failure, stroke, death, and dementia (2, 3). The etiology of AF is complex and can involve multiple systemic diseases. Various factors can increase the susceptibility to AF, including primary diseases cardiovascular diseases such as coronary heart disease, hypertension, valvular heart disease, cardiomyopathy, etc., endocrine diseases such as hyperthyroidism, respiratory diseases such as sleep apnea syndrome, and oxidative stress and other factors. Koray Kalenderoglu et al. (4) pointed out that in the development of AF, the inflammatory response is closely related to the onset and maintenance of atrial fibrillation. In addition, this study evaluated the levels of inflammatory markers after balloon ablation to predict the risk of AF recurrence. It was found that the inflammatory indexes, namely the uric acid/albumin ratio (UAR), the systemic immune—inflammation index (SIII), and the CRP/albumin ratio (CAR), were independently associated with the risk of AF recurrence after balloon ablation. Moreover, it was discovered that CAR might be better at predicting the recurrence of AF.

Chronic kidney disease (CKD) is a chronic disease with abnormal kidney structure or function lasting for more than 3 months. The key points of its common diagnostic criteria are as follows (5): the appearance of albuminuria (UAER ≥ 30 mg/24 h or UACR ≥ 30 mg/g), abnormal urinary sediment, related tubular lesions, histological abnormalities, structurally abnormal findings in imaging, or a history of kidney transplantation, etc. CKD is a disease that seriously threatens human health, apart from cardiovascular and cerebrovascular diseases, diabetes, malignant tumors, and other diseases. In recent years, the prevalence of CKD has been on the rise globally, and the prevalence rate in the general population has reached as high as 14.3% (6, 7). In the development process of chronic kidney disease, inflammatory responses play an indispensable role (8). Some studies have pointed out that in the early stage of kidney injury, neutrophils, the main recruited inflammatory cells, promote the production of reactive oxygen species (ROS) and trigger an inflammatory cascade reaction (9). The inflammatory response, as an important immune defense mechanism of the body, is a dynamic process of injury, anti-injury, and repair, and can lead to the occurrence of various chronic diseases.

It was first discovered by Robert Bright and others in 1,836 that there is an association between the physiology and pathology of the heart and the kidney, and it was described as “the delicate and highly interdependent relationship between the heart and the kidney”. With the continuous advancement of research on the heart and the kidney, a new concept- “Cardiorenal Syndrome (CRS)” (10, 11) has been proposed, which refers to a syndrome in which acute or chronic functional abnormalities occur in one of the heart and kidney organs, resulting in acute or chronic functional abnormalities in the other organ. The heart and kidney are both important organs that affect hemodynamics, and at the same time, they are both affected by hemodynamics, and there is a strong interaction between them (12). The pathogenesis of cardiorenal syndrome involves multiple aspects, including changes in hemodynamics, enhanced activity of the renin-angiotensin-aldosterone system (RAAS) (13), oxidative stress, production of advanced glycation end-products, endoplasmic reticulum stress, and persistent chronic inflammation, etc., and its exact mechanism has not been fully elucidated. At present, regarding the pathophysiological mechanisms of AF and CKD, many studies have identified that chronic inflammatory responses play important roles in the occurrence and development of these two diseases. This study's purpose is to analysis the potential biological significance of biomarkers in AF and CKD, further investigate the immune characteristics of immune cells in these two diseases, and search for potential diagnostic markers and therapeutic targets.

## 2 Materials and methods

### 2.1 Acquisition of datasets

**Figure 1:**
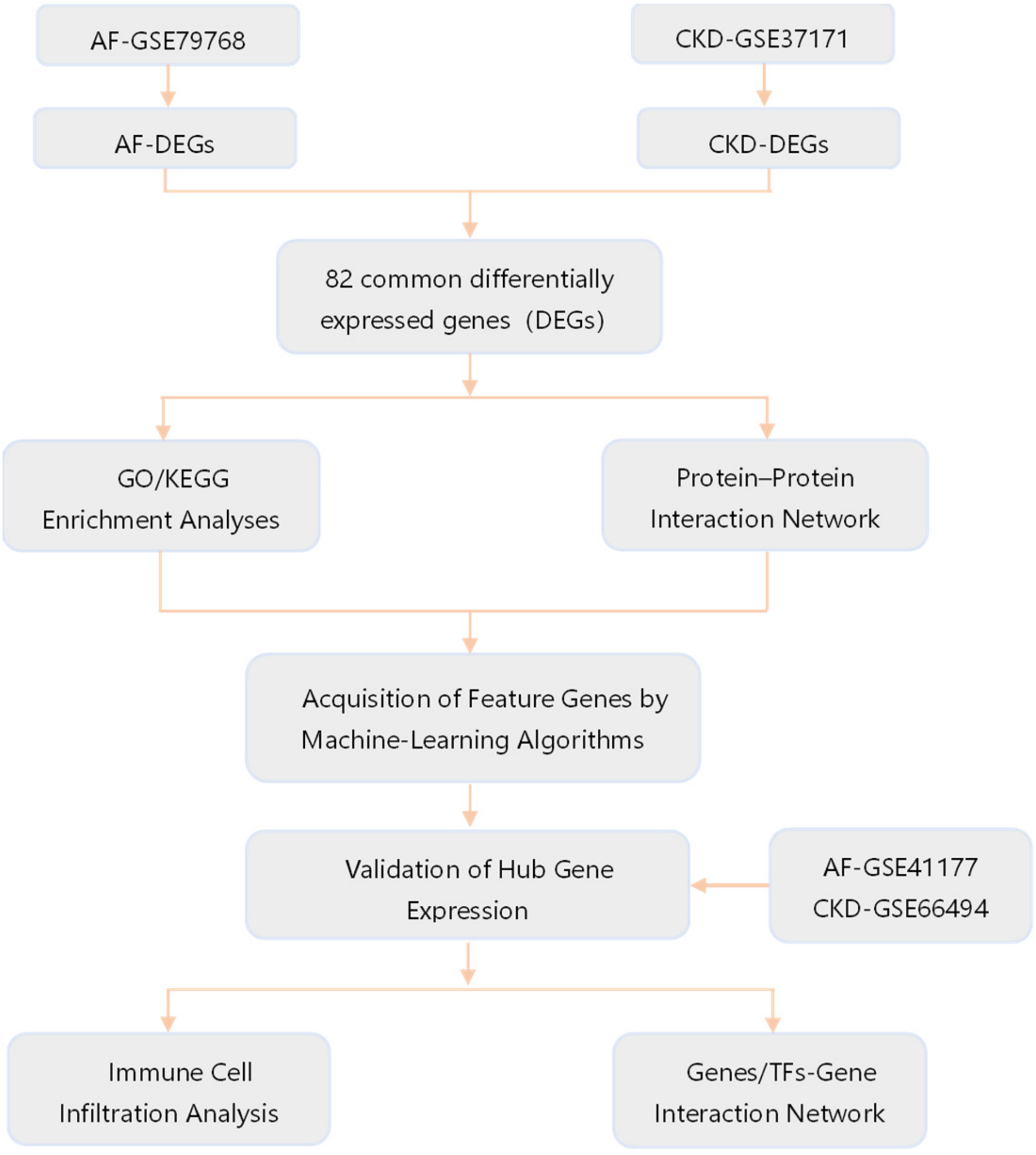
Flow chart of the study design. GO, gene ontology; KEGG, Kyoto encyclopedia of genes and genomes; TFs, transcription factors.

The gene expression profile datasets of AF and CKD were downloaded from the Gene Expression Omnibus (GEO, https://www.ncbi.nlm.nih.gov/geo/). Among them, GSE79768 and GSE14975 are AF-related datasets, with a total of 19 atrial fibrillation case groups and 17 sinus rhythm control groups. GSE37171 and GSE120683 are CKD-related datasets, with a total of 78 chronic kidney disease patients and 43 normal control groups. Among them, the AF dataset GSE14975 and the CKD dataset GSE120683 are used as external validation sets, and all of the above datasets are obtained from the GPL570 platform. The specific contents of the gene chip data of the two diseases are shown in Table 1, and the research analysis process is shown in Figure 1.

**Table 1:** Basic information of GEO datasets used in the study.

GSE series	Platform	Samples size		Disease
		Control	Disease	
GSE79768	GPL570	12	14	AF
GSE37171		40	75	CKD
GSE14975		5	5	AF
GSE120683		3	3	CKD

### 2.2 Preliminary data processing and DEGs screening

**Figure 2:**
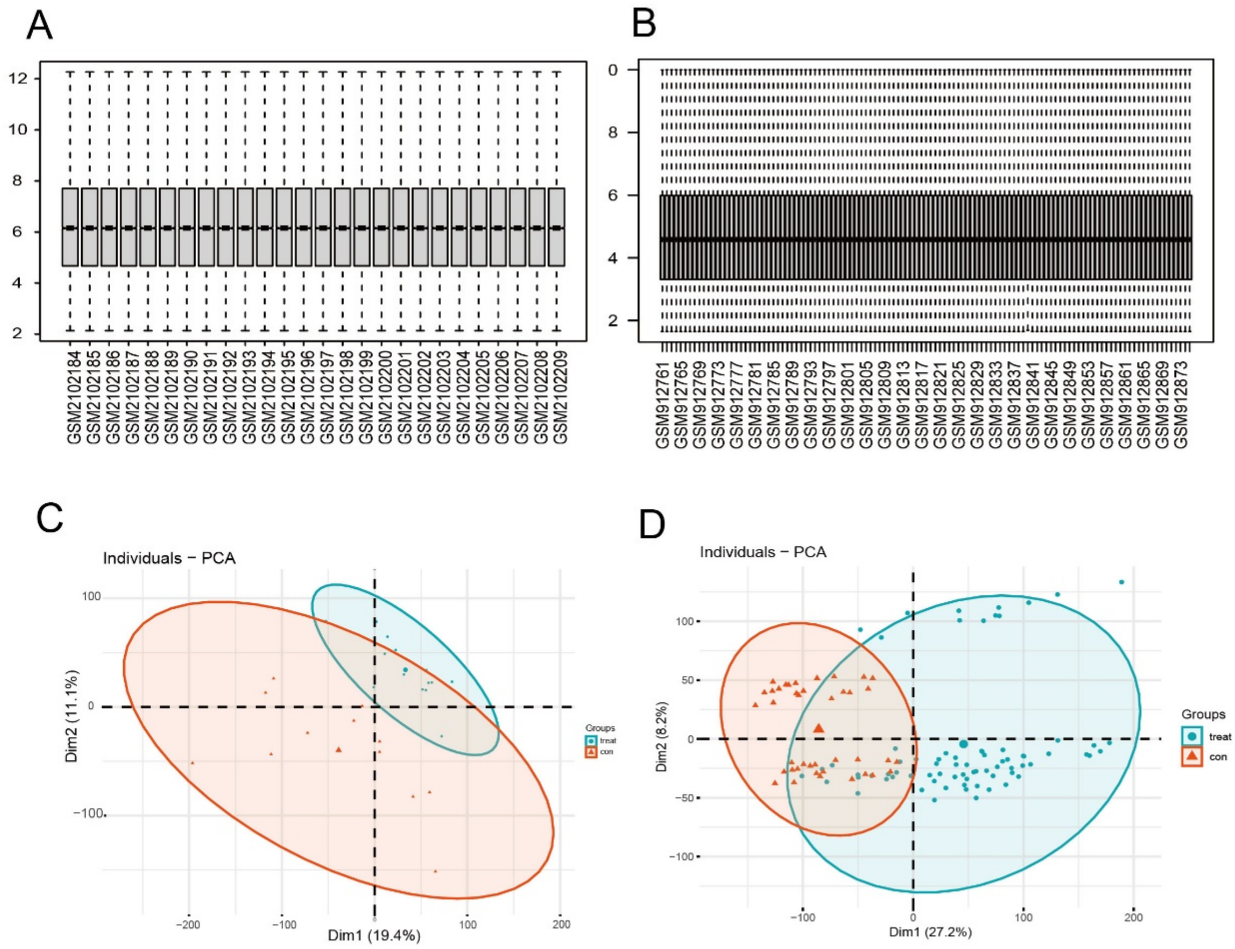
Normalized expression matrices. Box plots **(A,B)** and PCA diagrams **(C,D)** of the GSE79768 and GSE37171 datasets. PCA, principal component analysis.

The downloaded microarray datasets are read in based on the R (4.4.1) software. The expression data of the selected samples are annotated according to the GPL570 platform file, and the probe ID conversion is carried out to transform the probe matrix into a gene matrix. When a probe corresponds to multiple gene names, the first gene in the front is retained through the delimiter. The data are corrected by using the Normalize Between Arrays function of the “limma” package. The box-plots of the samples after standardizing the original data are shown in Figures 2A,B, respectively; the PCA plots after performing principal component analysis are shown in Figures 2C,D. Obtain the grouping information of the datasets of the two diseases. Use the “limma” package in R software to conduct differential expression analysis on the gene expression profile matrices of AF and CKD respectively. Sequentially, through constructing a comparison matrix, fitting a linear model, and performing Bayesian testing, all the differentially expressed genes (DEGs) of the two diseases are finally obtained. To further screen the DEGs, for both the gene data of AF and CKD, *P* < 0.05 and |log2(FC)| > 0.585 are selected as the screening thresholds. With the help of the “pheatmap” package, heatmaps and volcano plots are drawn for the two diseases based on the obtained data. Furthermore, use the “VennDiagram” package in R software to draw a Venn diagram to identify the overlapping part of the two sets of DEGs, and obtain the co-expressed differentially expressed genes of the two groups of disease data. This overlapping part of co-expressed DEGs is used for subsequent research and analysis.

### 2.3 Gene ontology (GO) and Kyoto encyclopedia of genes and genomes (KEGG) enrichment analyses

Utilize the “org.Hs.eg.db” R package for human genome annotation to transform the gene names in the co-expressed DEGs data of the two diseases, which is obtained from the above mentioned differential analysis, into ENTREZ IDs. By means of the “ClusterProfiler” package in R software, conduct GO and KEGG enrichment analyses on the co-expressed DEGs. Meanwhile, the enrichment analyses need to meet the conditions: *p. adjust* <0.05 and *q. value* <0.05. The results of gene function enrichment analyses are visualized through the “ggplot2”, “pathview” and “enrichplot” R packages. Finally, using the “cnetplot” function of the “BiocManager” R package to screen out the Top4 results in GO analysis and the Top8 results in KEGG analysis, and visualizing the results.

### 2.4 Construction of protein—protein interaction (PPI) network

The co-expressed differentially expressed genes obtained after taking the intersection are imported into the STRING (https://cn.string-db.org) website, and the confidence score threshold is set to 0.4 to evaluate the interactions between genes by constructing a protein-interaction network diagram, and the screened results are downloaded. Then, with the help of Cytoscape software, the results obtained from the STRING database are visualized, and the top 15 genes are screened based on the topological algorithm-Matthews correlation coefficient (MCC).

### 2.5 Acquisition of feature genes by machine-learning algorithms

The top 15 co-expressed genes obtained by the MCC algorithm are further screened for key feature genes in AF and CKD respectively through two machine-learning algorithms. The least absolute shrinkage and selection operator (LASSO) and random forest (RF) algorithms are used to obtain the key feature genes of the two diseases respectively, and the key feature genes of the two diseases are obtained by taking the intersection through Venn diagrams.

### 2.6 Verification of gene diagnostic efficacy with external datasets

The diagnostic values of the key characteristic genes obtained by machine-learning algorithms in the two diseases are evaluated by using the area under the ROC curve (AUC). The value of AUC ranges from 0.5 to 1. The closer it is to 1, the better the diagnostic performance and the higher the accuracy of the gene. The AF dataset GSE14975 (AF = 5, SR = 5) and the CKD dataset GSE120683 (CKD = 3, Normal = 3) are downloaded from the GEO database. The ROC curves of the key characteristic genes in the two diseases are drawn with the help of the “pROC” and “ggplot2” packages, and the AUC values and 95% CI values are calculated to verify the diagnostic efficacy of the key genes in the two diseases. Draw a violin plot using the t-test to verify the diagnostic value of key characteristic genes in the training sets GSE79768 and GSE37171.

### 2.7 Construction of gene-gene and TFs-gene interaction network diagrams

The gene-gene interaction network of biomarkers, that is, key characteristic genes, is analyzed with the help of the GeneMANIA online database; based on the JASPAR database (https://jaspar.genereg.net/), the transcription factors (TFs)-gene regulatory network is constructed using the Networkanalyst platform, and the key transcription factors are found by using the cytohubba plug-in MCC algorithm of Cytoscape software.

### 2.8 Immune cell infiltration analysis

The expression data of 82 co-expressed differentially expressed genes in the atrial fibrillation validation set GSE79768 and the chronic kidney disease validation set GSE37171 were obtained. The infiltration abundances of immune cells in the two disease validation sets were evaluated based on the “CIBERSORT” R package. The results were displayed by drawing bar charts, box plots, and heat maps using ggplot2. The correlation coefficients and *P—values* between the target genes and immune cell subsets were calculated using the “cor.test” function, and the analysis results were visualized with lollipop plots.

## 3 Results

### 3.1 Screening and identification of DEGs of the two diseases

**Figure 3:**
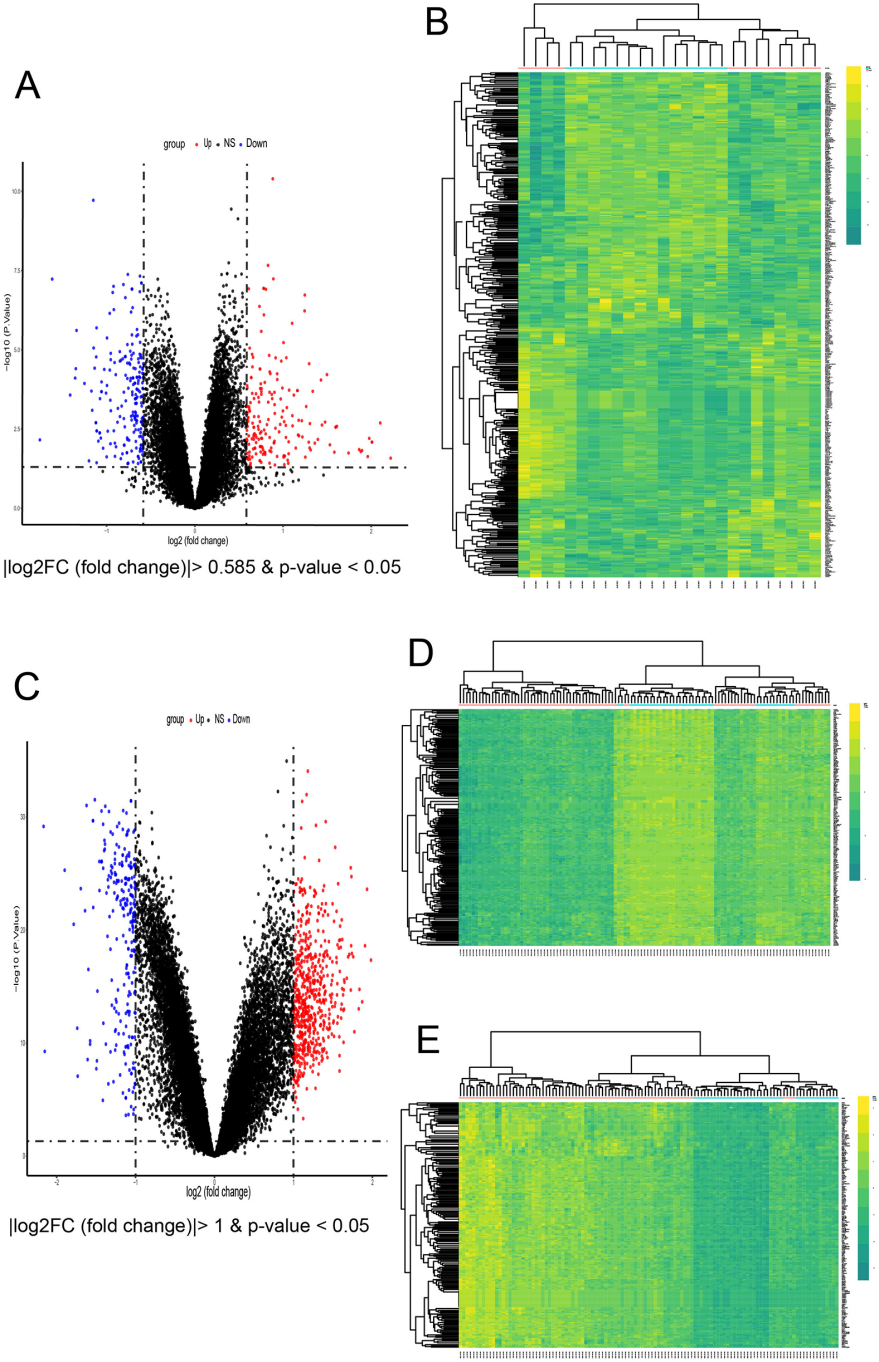
Identification of common DEGs and enrichment analysis. **(A)** Volcano plots of all DEGs in GSE79768. **(B)** Heatmap of all DEGs in GSE79768. **(C)** Volcano of all DEGs in GSE37171. **(D,E)** The top 200 up-regulated concurrently with the top 200 down-regulated DEGs of in GSE37171.

**Figure 4:**
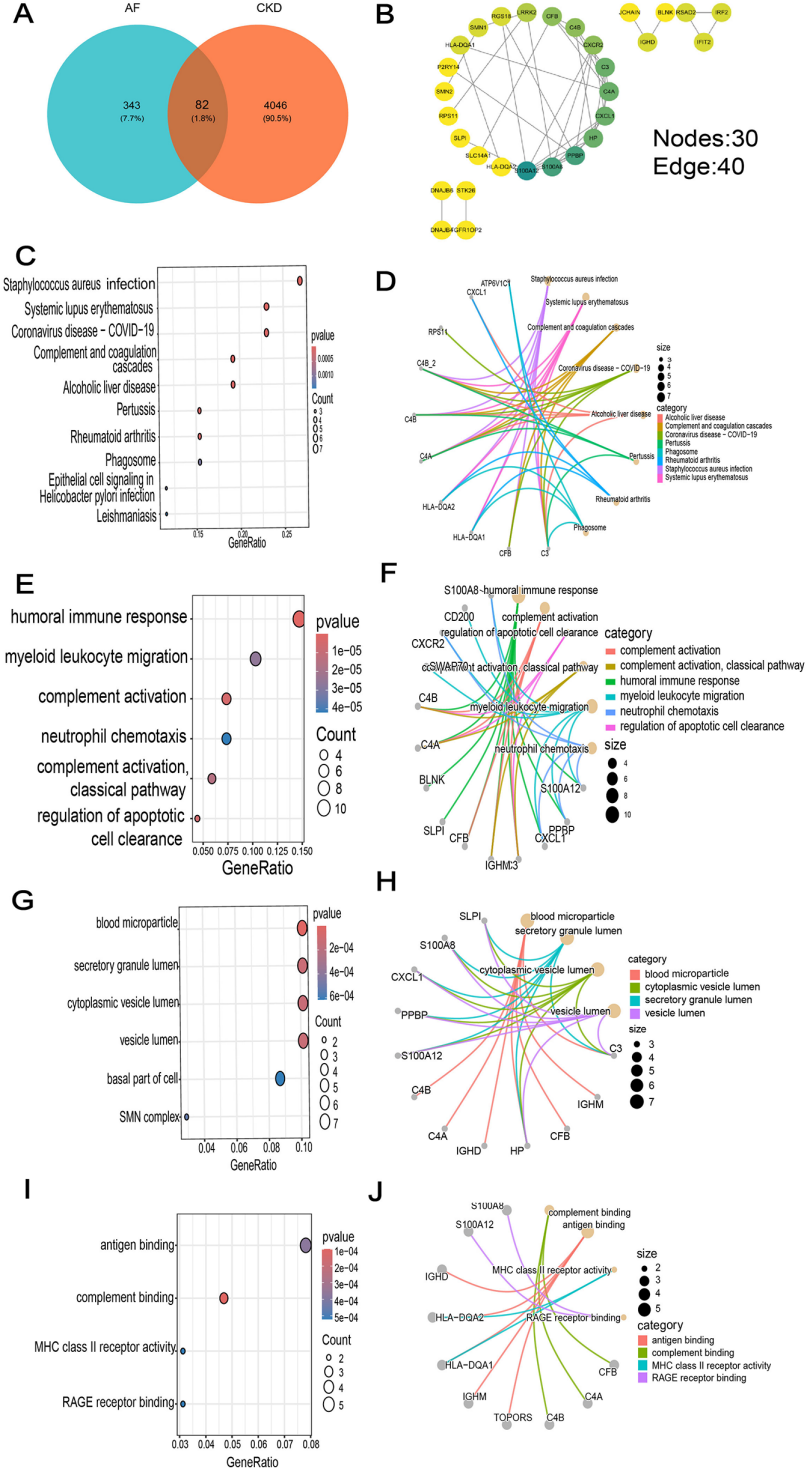
GO and KEGG enrichment analysis of common DEGs. **(A)** Venn diagram of common DEGs between AF and CKD. **(B)** The PPI network diagram beautified with the help of Cytoscape software. **(C)** Top 10 results in KEGG pathway enrichment analysis of the common DEGs. **(D–J)** The top 8 KEGG pathways **(D)** and the top 4 GO (BP, CC and MF) terms **(E–J)** of the common DEGs and their enriched targets were identified.

In the AF dataset GSE79768, which contains 14 atrial fibrillation patients and 12 sinus—rhythm normal control groups, a total of 425 DEGs were screened (Figures 3A,B), among which 210 genes were up-regulated and 215 genes were down-regulated. In the CKD dataset GSE37171, which contains 75 chronic kidney disease patients and 40 normal control groups, a total of 4,128 DEGs were screened, among which 660 genes were up-regulated and 285 genes were down-regulated [with threshold conditions: *P-value* < 0.05 and |log2(FC)| > 1, which were illustrated by the volcano plots in Figures 3C–E]. 82 co expressed DEGs were obtained by taking the intersection of the DEGs of the two diseases through Venn diagrams (Figure 4A).

### 3.2 GO/KEGG enrichment analyses of co-expressed DEGs

In the results of GO enrichment analysis, from the perspective of biological processes, the genes were mainly enriched in humoral immune responses, lymphocyte-mediated immunity, adaptive immune responses of immunological receptor somatic recombination based on the immunoglobulin superfamily domain, and myeloid leukocyte migration (Figures 4E,F). From the perspective of cellular composition, the genes were mainly enriched in secretory granule lumen, blood microparticles, cytoplasmic vesicle lumen, and vesicle lumen (Figures 4G,H). From the perspective of molecular functions, the genes were mainly enriched in complement binding, antigen binding and MHC class II receptor activity (Figures 4I,J). KEGG functional enrichment analysis indicated that the genes were mainly enriched in Staphylococcus aureus infection, systemic lupus erythematosus, COVID-19, alcoholic liver disease, and the complement—coagulation cascade process (Figures 4C,D).

### 3.3 Constructing a PPI network to obtain candidate genes

**Figure 5:**
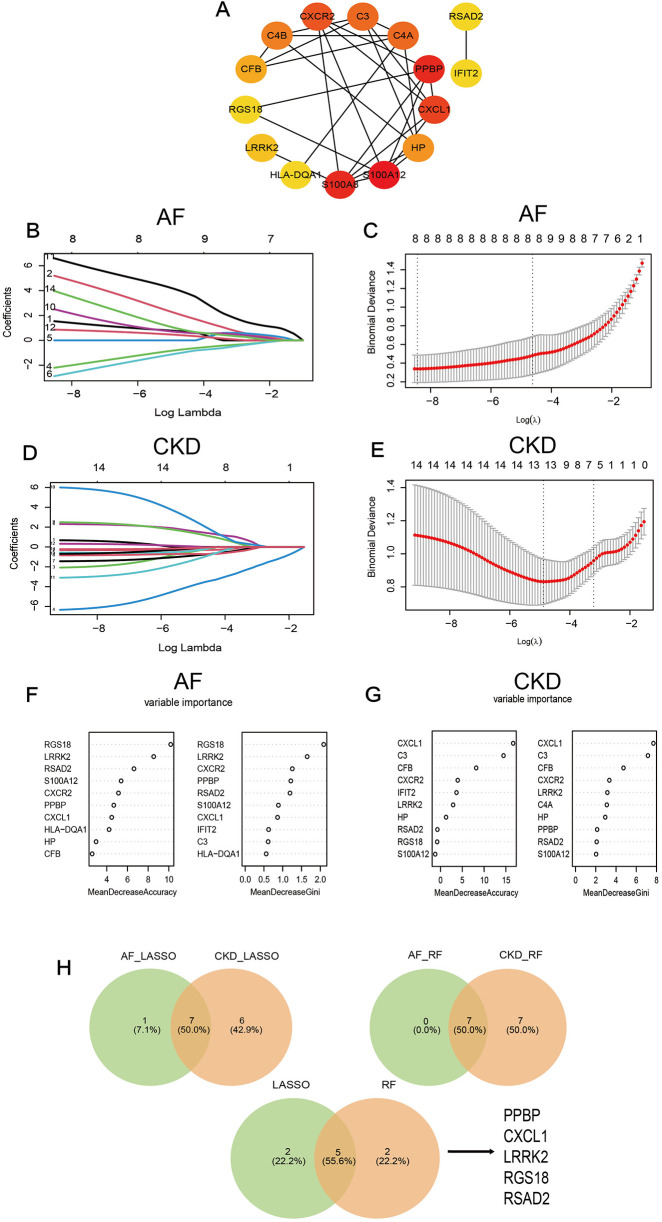
**(A)** The PPI diagram of the TOP 15 genes screened by the MCC algorithm. **(B–E)** LASSO regression analysis for screening in the hub genes in GSE79768 and GSE37171, cross-validation was performed to select the best ⅄, and the lines in the regression coefficient path map representative the variables included and their running trajectories. **(F,G)** Screen biomakers for hub genes in GSE79768 and GSE37171 using the RF algorithm. **(H)** The Venn diagram identified 5 candidates for hub genes by LASSO regression analysis and RF algorithm.

A PPI network of 79 co-expressed genes was constructed using the STRING online website. After removing the isolated nodes, it was imported into Cytoscape software to obtain a PPI network diagram with 30 nodes and 40 edges (Figure 4B), and the top 15 genes were obtained based on the MCC algorithm (Figure 5A).

### 3.4 Feature genes are obtained by machine-learning algorithms

Further screening was performed on the top 15 genes of AF and CKD respectively based on two machine-learning algorithms (LASSO algorithm and RF algorithm). After gene screening for AF (Figures 5B,C) and CKD (Figures 5D,E) by the LASSO algorithm respectively, the intersection was taken to obtain 7 characteristic genes: PPBP, CXCL1, C3, LRRK2, RGS18, HLA—DQA1, RSAD2. When applying the RF algorithm for gene screening, with the criterion of Mean Decrease Gini >0.8 or 1 set, analyses were carried out for AF (Figure 5F) and CKD (Figure 5G) respectively, and the intersection of the two obtained through a Venn diagram yielded 7 characteristic genes: PPBP, CXCL1, CXCR2, LRRK2, RGS18, RSAD2, S100A8. Then, the characteristic genes obtained by the two machine-learning algorithms were intersected, and finally, 5 key characteristic genes were obtained: PPBP, CXCL1, LRRK2, RGS18, RSAD2 (Figure 5H).

### 3.5 Verification of gene diagnostic efficacy with external datasets

**Figure 6:**
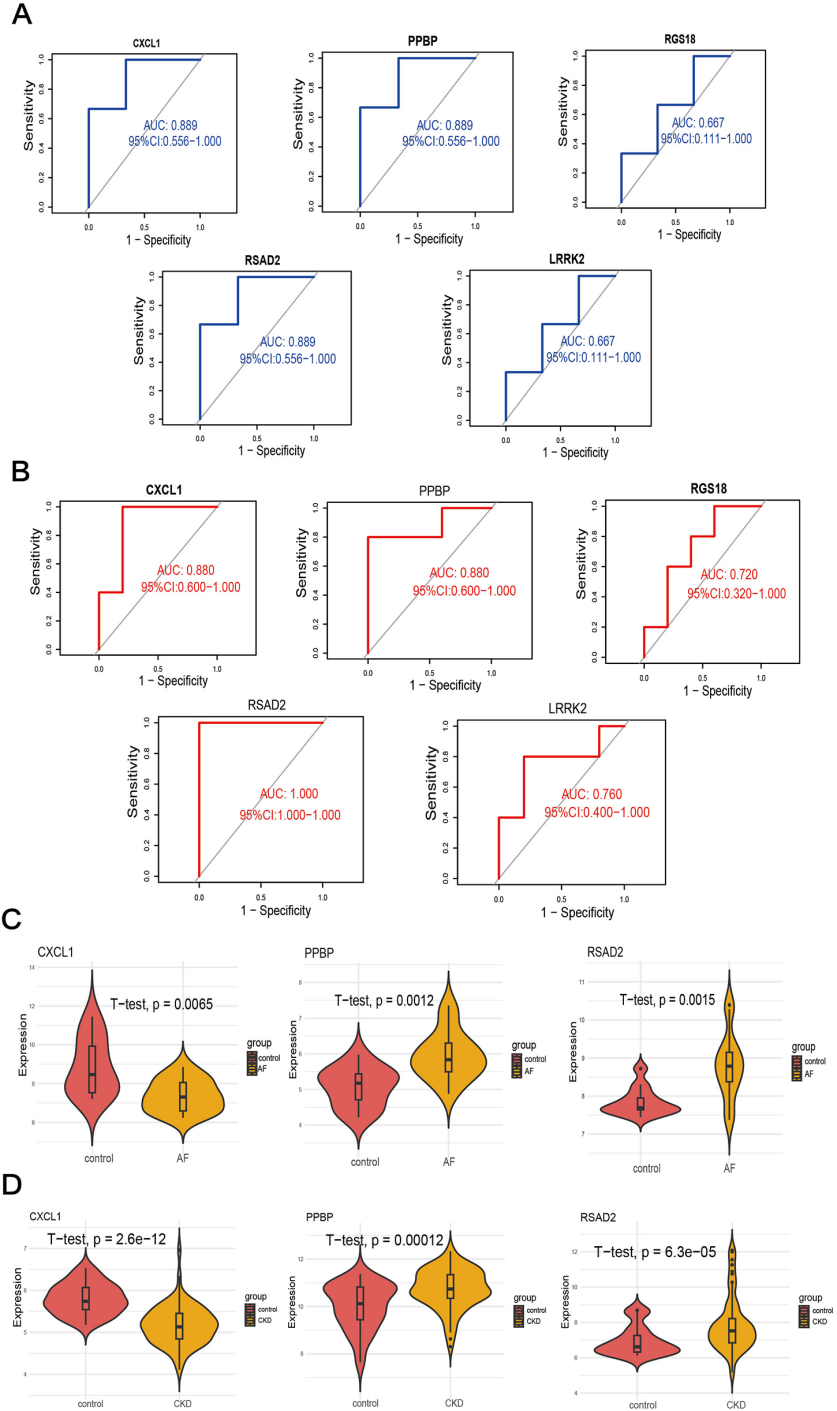
Validation of candidate genes. **(A,B)** ROC curves of candidates genes to assess for diagnostic sensitivity and specificity value in GSE14975 **(A)** and GSE120683 **(B)**. **(C,D)** Validation of the diagnostic efficacy of candidate genes in the GSE79768 **(C)** and GSE37171 **(D)** respectively by *T*-test.

ROC curves were constructed for the five key characteristic genes in the external validation datasets respectively, and the diagnostic efficacy of the characteristic genes for diseases was verified by calculating the area under the curve. Verification in the AF dataset GSE14975 found that RSAD2 (AUC = 1.000, 95%CI = 1), CXCL1 (AUC = 0.880, 95%CI = 0.626–1), LRRK2 (AUC = 0.760, 95%CI = 0.4138–1), PPBP (AUC = 0.880, 95%CI = 0.626–1), and RGS18 (AUC = 0.720, 95%CI = 0.365–1), among which RSAD2 had the highest diagnostic value for AF (Figure 6A). Verification in the CKD dataset GSE120683 showed that RSAD2 (AUC = 0.889, 95%CI = 0.5809–1), CXCL1 (AUC = 0.889, 95%CI = 0.5809–1), LRRK2 (AUC = 0.667, 95%CI = 0.1332–1), PPBP (AUC = 0.889, 95%CI = 0.5809–1), and RGS18 (AUC = 0.667, 95%CI = 0.1332–1), among which RSAD2, CXCL1, and PPBP had the same diagnostic value for CKD (Figure 6B). Therefore, three co-expressed biomarkers of the two diseases were obtained based on AUC > 0.7: PPBP, CXCL1, and RSAD2. In the two training sets of AF GSE79768 (Figure 6C) and CKD GSE37171 (Figure 6D), violin plots were drawn, and the results showed that the biomarkers had good diagnostic value (*p* *<* *0.05*).

### 3.6 Construction of genes-genes and TFs-genes interaction network diagrams

**Figure 7:**
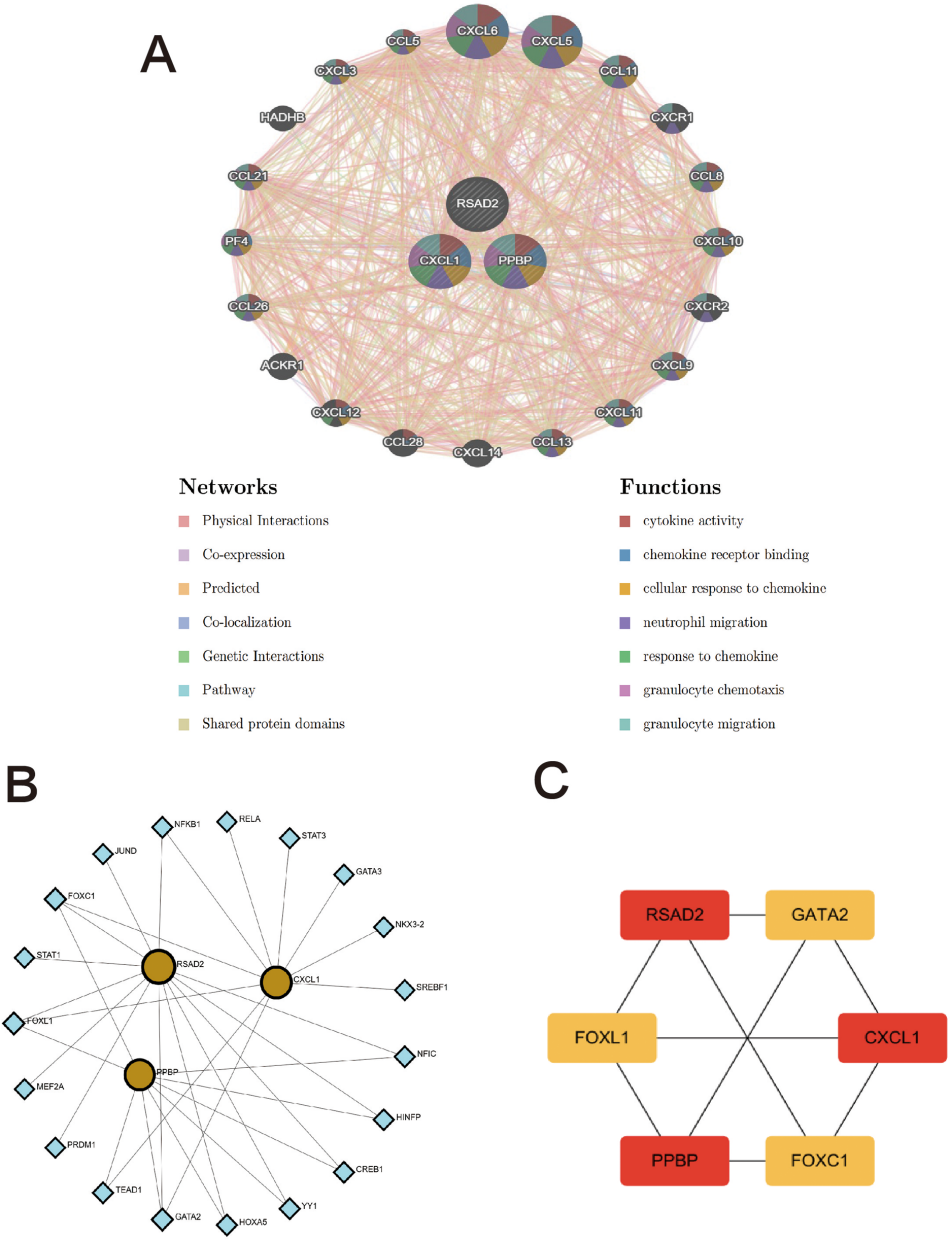
The interaction network of hub genes. **(A)** Three hub genes were analyzed via GeneMANIA. The 20 most frequently changed neighboring genes are shown. The predicted genes are located in the outer circle and hub genes are in the inner circle. **(B)** TFs-gene network analysis of hub genes was displayed via Networkanalyst. The yellow located within the circle represents hub genes, and the blue located on the periphery represents TFs that are closely related to hub genes. **(C)** Screening key transcription factors by MCC algorithm. The red represents hub genes, and the yellow represents TFs.

The Genes-Genes interaction network of biomarkers was analyzed using the GeneMANIA database. The protein-interaction relationships involved in the three biomarkers are shown in Figure 7A, displaying the adjacent genes that change most frequently with the biomarkers. PPBP, CXCL1, and RSAD2 have complex interaction networks, and they interact with the surrounding shared genes through co-expression, physical interaction, prediction, genetic interaction, co-localization, pathways, and shared protein-domain interaction. Among them, the two biomarkers BBPB and CXCL1 mainly have functional interactions with cytokine activity, chemokine receptor activity, cell response to chemokines, neutrophil migration, response to chemokines, granulocyte chemotaxis, and granulocyte migration. The above results once again emphasize the importance of immune-cell-related inflammation in these two diseases, and PPBP and CXCL1 may be key regulatory genes in the development mechanisms of the two diseases.

The TFs-genes interaction network was constructed using the Networkanalyst platform to find potential regulatory molecules (Figure 7B), and the key transcription factors were found using the cytohubba plug-in MCC algorithm. Finally, it was confirmed that FOXC1, FOXL1, and GATA2 are the main transcription factors of the key characteristic genes (Figure 7C).

### 3.7 Correlation analysis between key characteristic genes and immune cell infiltration

**Figure 8:**
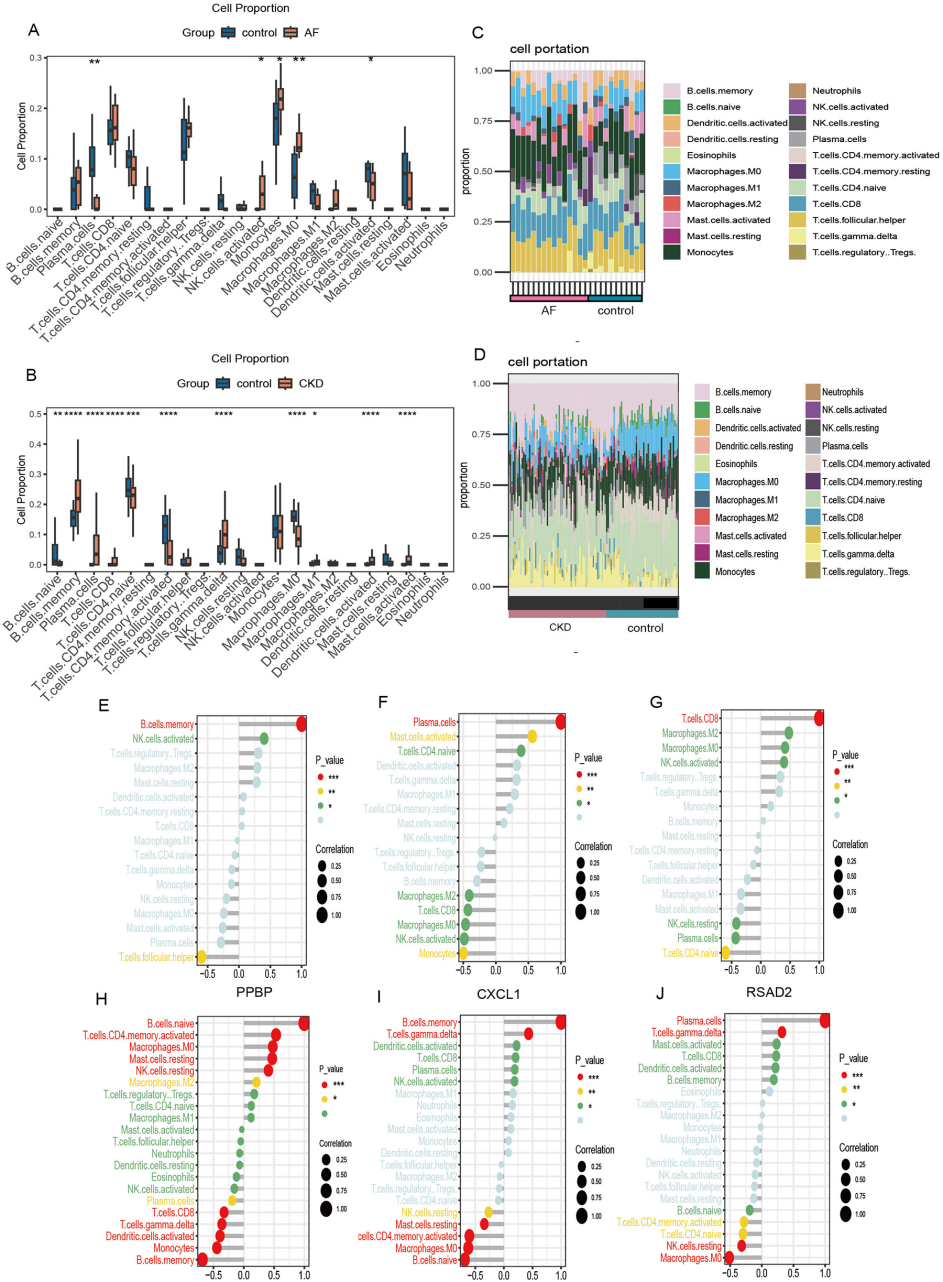
Immune cell infiltration. **(A,B)** Box plots show the comparison of 22 types of immune cells between the disease group and the control group of GSE79768 and GSE37171. **(C,D)** Immune cell infiltration maps of each sample of GSE79768 and GSE37171. **(E–G)** The correlations between the expression of three hub genes (PPBP, CXCL1, RSAD2) and immune cell in GSE79768. **(H–J)** The correlations between the expression of three hub genes (PPBP, CXCL1, RSAD2) and immune cell in GSE37171. (*p < 0.05, **p < 0.01, ***p < 0.001, ****p < 0.0001).

Enrichment analysis of co-expressed genes showed that the genes were mainly enriched in pathways such as the complement- coagulation cascade and systemic lupus erythematosus, indicating that the genes are closely related to immune-inflammatory responses. To further explore the infiltration differences of various immune cells in the case and control groups of the two diseases respectively, the proportions of each sample of the two diseases in 22 kinds of immune cells were obtained based on the “CIBERSORT” algorithm (Figures 8A-D). The results showed that compared with healthy controls, the common feature of AF and CKD is a consistent upward trend of B memory cells and CD8+ T cells. In the AF dataset, compared with the control group, the AF case group was significantly up-regulated in monocytes, M0 macrophages and activated natural killer (NK) cells (*p* *<* *0.05*), and significantly down-regulated in activated dendritic cells and plasma cells (*p* *<* *0.05*). In the CKD dataset, the CKD case group was significantly up-regulated in B memory cells, γ-δ T cells, plasma cells, activated mast cells, M1 macrophages, activated dendritic cells, and CD8+ T cells (*p* *<* *0.05*), and significantly down-regulated in resting CD4+ T cells, M0 macrophages, activated CD4 + memory cells, and resting B cells (*p* *<* *0.05*).

To further investigate the association between biomarkers and immune cells, Spearman rank analysis was carried out. In the figure, the horizontal axis represents the correlation between biomarkers and immune cells, with dots representing the size of the correlation coefficient. The larger the dot, the greater the correlation. The vertical axis represents the types of immune cells, and the number of asterisks indicates the size of the *P*-value. The smaller the *P*-value, the more asterisks.

The results of the correlation analysis show that in the AF data, PPBP is significantly positively correlated with B memory cells and activated NK cells, and significantly negatively correlated with follicular helper T cells (Figure 8E); CXCL1 is significantly positively correlated with plasma cells, activated mast cells, and resting CD4+ T cells, and significantly negatively correlated with monocytes, activated NK cells, CD8+ T cells, M0- and M2- type macrophages (Figure 8F); RSAD2 is significantly positively correlated with CD8+ T cells, activated NK cells, M0- and M2- type macrophages, and significantly negatively correlated with resting CD4+ T cells, plasma cells, and resting NK cells (Figure 8G). In the CKD data, PPBP is significantly positively correlated with resting B cells, activated CD4 + T memory cells, resting mast cells, resting NK cells, M0- and M2- type macrophages, and significantly negatively correlated with B memory cells, monocytes, activated dendritic cells, γ-δ T cells, CD8+ T cells, and plasma cells (Figure 8H); CXCL1 is significantly positively correlated with memory B cells and γ-δ T cells, and significantly negatively correlated with resting B cells, M0- type macrophages, activated CD4+ T memory cells, resting mast cells, and resting NK cells (Figure 8I); RSAD2 is significantly positively correlated with plasma cells and γ-δ T cells, and significantly negatively correlated with M0- type macrophages, resting NK cells, activated CD4+ T memory cells, and resting CD4+ T cells (Figure 8J).

## 4 Discussion

At present, an increasing number of studies have shown that the systemic inflammatory response state and immune cells play an important role in the occurrence and development of AF and CKD. For example, mast cells promote the expression of cardiac fibroblast cells and collagen by secreting platelet-derived growth factor-A (PDGF A) (14). During heart injury, macrophages present antigens released by dead heart tissue cells to CD4 + T cells through protein molecules on their surface and activate them, leading to the continuous release of inflammatory factors and the continuous progress of pro-fibrosis, thereby expanding heart damage (15–17). When the kidneys are damaged, it causes the continuous release of inflammatory factors, resulting in continuous damage to kidney tissue and function, thereby leading to CKD. Some studies have pointed out after ureteral ligation in wild-type mice, disodium cromoglycate, a mast cell stabilizer that reduces degranulation, significantly alleviated renal fibrosis. This conclusion is consistent with the immune infiltration results of CKD disease in this study (18). Similarly, both AF and CKD are two diseases with a high risk of thrombosis. Both are associated with an increased risk of thrombosis and embolism events. The process of thrombosis involves the participation of various cytokines and coagulation factors, which play important roles. A study exploring a mouse model found that edoxaban significantly inhibited the levels of inflammatory factors, upregulated the levels of chemokines, and reduced the risks of apoptosis, atrial fibrillation, and venous thrombosis by regulating the Wnt-β-mediated PI3K/AKT signaling pathway (19). In this study, through KEGG enrichment analysis, it was found that the differentially expressed genes were enriched in the complement—coagulation cascade process, which is consistent with the research findings. In addition, we found that CXCL1 and PPBP are closely associated with CXCR2 (C-X-C motif chemokine receptor 2). As a chemokine receptor, CXCR2 can mediate apoptosis, inflammatory responses, and angiogenesis through pathways such as the phosphatidylinositol 3-kinase/Akt (PI3K/Akt) pathway, the phospholipase C/protein kinase C (PLC/PKC) pathway, and the NF -κB pathway. These processes are closely related to the mechanism of thrombosis.

In our study, we attempted to identify DEGs and deeply explore the role of immune cell infiltration in AF-CKD. A total of 82 DEIRGs were detected as candidate biomarkers in the pathogenesis of AF-CKD, and the enrichment function identified the potential mechanisms of DEIRGs. The results of KEGG enrichment analysis show that DEIRGs are significantly related to immune and inflammatory responses such as systemic lupus erythematosus, COVID-19, and the complement- coagulation cascade. In addition, through GO analysis, these genes are involved in humoral immune responses, lymphocyte- mediated immunity, adaptive immune responses of immunological receptor somatic recombination based on the immunoglobulin superfamily domain, secretory granule lumen, blood microparticles, cytoplasmic vesicle lumen, vesicle lumen, complement binding, antigen binding, and other pathways. The study found three potential biomarkers of AF and CKD-PPBP, CXCL1, and RSAD2, and the CXCLs/CXCR signaling pathway plays an important role in the occurrence and development of the two diseases.

CXCL1 (C-X-C motif chemokine receptor 1) is a member of the CXC superfamily and an important chemokine that participates in the occurrence and development of various inflammatory diseases, activating CXC chemokine receptor 2 (CXCR2) and high-level CXC chemokine receptor 1 (CXCR1). It has important functional roles in physiological functions, including inducing angiogenesis and neutrophil recruitment. In a 2018 study by Lei Wang et al. (20), the researchers found that angiotensin II-induced monocyte infiltration in the heart is mainly mediated by CXCL1-CXCR2 signaling, and CXCL1-CXCR2 signaling initiates and promotes cardiac remodeling. In addition, the study pointed out that intervention with CXCL1 neutralizing antibody prevents the development of angiotensin II-induced cardiac hypertrophy, fibrosis, and inflammation (21). As a pro- inflammatory chemokine, CXCL1 mediates various acute and chronic inflammations, thereby promoting fibrosis development. In particular, monocyte infiltration in atrial tissue leads to an increase in the number of macrophages in the atrium, and then macrophages can directly couple with surrounding cardiomyocytes through gap junctions and increase atrioventricular conduction by stimulating cardiomyocyte repolarization (22). The expression level of CXCL1 in the atrium of atrial fibrillation patients is strongly positively correlated with activated mast cells (23), which is consistent with the results of immune infiltration analysis in this study. Activated mast cells and macrophages (24), especially those interacting with vascular smooth muscle cells, produce CXCL1 in the intima and perivascular tissue. In AF patients (14), mast cells may induce cardiac fibroblasts to increase collagen production by secreting PDGF-A, resulting in atrial fibrosis and AF CXCL1 may mediate inflammatory responses through the NF-κB (nuclear factor-κB), TGF-Smad 2/3 signaling pathways, among which TGF-β is an important pro-fibrotic cytokine. Similarly, some studies have pointed out (25) that in damaged kidney cells, when TGF-β is continuously expressed, pathological accumulation of extracellular matrix substances in the glomerulus and interstitial compartments occurs, leading to the progression of renal fibrosis (26). Damaged renal tubules activate myofibroblast differentiation, proliferation, and matrix secretion by producing and releasing bioactive molecules to recruit inflammatory cells. A single-cell analysis pointed out (27) that CXCL1 is secreted from pro-fibrotic tubules and then recruits CXCR2 + basophils, which is a key factor in renal fibrosis. Some studies have found that the expression of TNF-α, and CXCL-1 increases in the kidneys of CKD rats. After treatment with tanshinone IIA, it was found that the drug inhibits kidney inflammation mediated by the NF-κB signal and reduces the expression of these inflammatory genes (26).

PPBP (pro-platelet basic protein), also known as CXCL7 or NAP2, belongs to the platelet—derived growth factors of the CXC chemokine family. It is an effective chemoattractant and activator of neutrophils. It is released from activated platelets and participates in the response to vascular injury. It can stimulate various processes, such as mitosis, glucose metabolism, and the synthesis of extracellular matrix and plasminogen activators. Some studies have pointed out (23) that CXCL7/PPBP and CXCL1 are the hub genes with the most associations in atrial fibrillation. It is now clear that PPBP plays a crucial role in coordinating neutrophil recruitment during vascular injury. Some studies have explored (28, 29) the molecular basis for chemokine CXCL7 to form heterodimers with chemokines CXCL1, CXCL4, and CXCL8, pointing out that these chemokines co-exist in platelet granules and are released upon platelet activation. By activating the CXCR2 receptor, they recruit and coordinate neutrophils to reach the site of tissue injury. The recruited neutrophils mediate microbial killing and initiate tissue repair. PPBP exerts the functions of recruiting neutrophils and inducing angiogenesis by activating the CXCR2 receptor and binding to sulfated glycosaminoglycans (sGAGs). However, continuously activated neutrophils further aggravate the initial injury, leading to various thrombosis-related cardiovascular and inflammatory diseases (30–33). An article published in 2013 pointed out (34) that there is a CXCL7 chemotactic gradient within the thrombus, which is indispensable for the migration of neutrophils within the thrombus. In addition, the study (35) showed that in the context of platelet-neutrophil communication, the ability of CXCL7 to exist as both a monomer and a dimer, as well as in both free and GAG-bound states, may maximize repair and reduce tissue injury and the occurrence of cardiovascular diseases. CXCL7 is unique.

RSAD2 (radical S-adenosyl methionine domain containing 2), also known as Viral inhibitory protein, Endoplasmic reticulum—associated, interferon—inducible (VIPERIN), is an antiviral gene. In a recent study by Örebro University (36), publicly available human carotid atherosclerosis data were used and experimental research was carried out. The results showed that the RSAD2 gene has a higher expression level in plaques. Further experimental research found that when human—derived aortic smooth muscle cells are stimulated by interferon—γ, knocking down the RSAD2 gene will lead to a decrease in the expression and secretion of CXCR3 chemokines CXCL9, CXCL10, and CXCL11, thereby reducing the chemotactic effect on monocytes and decreasing the migration of monocytes to the lesion site. During the process of atherosclerosis, RSAD2 may play a role through inflammatory and immune responses. In addition, studies have shown (37) that functional chemokine receptors, including CXCR3, are expressed in glomerular podocytes, and the activation of these receptors may induce the release of oxygen free radicals, leading to podocyte injury and the development of proteinuria. Currently, there are relatively few studies on RSAD2, and more research is needed to further explore the potential role of this gene in various diseases.

In the studies of the three biomarkers, it is summarized that PPBP, CXCL1, and RSAD2 have similarities in the disease development process. All of them can lead to the up- regulated CXCLs inducing, activating, and binding to CXCR chemokines, promoting the rapid recruitment of leukocytes in the inflammatory environment. Therefore, the CXCLs/CXCR signaling pathway may play an important role in the development of AF and CKD. The research on the CXCLs/CXCR signaling pathway in inflammatory diseases can involve many different systems, including the respiratory system, digestive system, cardiovascular system, and nervous system. The development of diseases in different systems is inseparable from extensive leukocyte infiltration. After receiving upstream signaling, chemokine receptors recruit leukocytes to gather at the inflammatory foci. However, leukocyte disorders and excessive infiltration lead to the destruction of organ structures, resulting in inflammation- related diseases. Among them, CXCR2, a chemokine receptor closely related to PPBP and CXCL1 in the development of AF and CKD, is a G-protein-coupled receptor (GPCR) and plays an important role in the human immune system, especially in cell chemotaxis, inflammatory responses, and tumorigenesis. Binding to CXCR2 can activate multiple G- protein—mediated signal cascade pathways, including the PI3K/Akt pathway, PLC/PKC pathway, mitogen-activated protein kinase/p38 (MAPK/p38) pathway, and Janus kinase 2/signal transducer and activator of transcription (JAK2/STAT3) pathway. These signal transduction pathways are several key intracellular signal transduction routes, and they play important roles in cell metabolism, apoptosis, inflammatory responses, angiogenesis, cytokine signaling, and immune responses (38). Different from PPBP and CXCL1, the expression of RSAD2 is related to CXCR3 chemokines. CXCR3 is one of the G-protein-coupled receptors, mainly binding to ligands such as CXCL9, CXCL10, and CXCL11, and participating in the chemotaxis of immune cells.

In addition, this study constructed a TFs-DEGs interaction network for further analysis of possible therapeutic targets. In the TFs-DEGs interaction network, FOXC1, FOXL1, and GATA2 were identified as key transcription factors. FOXC1 and FOXL1 transcription factors both belong to the “C” subgroup of the Forkhead box (FOX)/Winged helix family. FOX transcription factors play important roles in various biological processes, including cell proliferation, differentiation, survival, and death. FOX transcription factors are distributed in different tissues and cell types of the lungs, intestines, and cardiovascular system (39). They are involved in cell proliferation, autophagy, inflammation, and the differentiation of specific gene transcription during the development and adulthood of cardiovascular tissues (40, 41). In a human heart tissue experimental study (42), it was found that compared with the control group of human- induced pluripotent stem-cell-derived cardiomyocytes with normal telomere length (nTL-CMs), human-induced pluripotent stem-cell-derived cardiomyocytes with short telomeres (sTL-CMs) exhibited an increase in aging markers, a decrease in mitochondrial respiratory reserve, and contractile dysfunction. In addition, the study found that overexpression of FOXC1 in nTL-CMs fully induces myocardial aging, induces inflammation—related genes, and simultaneously down- regulates myofibrillar and mitochondrial genes, leading to contractile dysfunction and myocardial aging. Conversely, knockdown of shFOXC1 in sTL- CMs is sufficient to block aging and restore mitochondrial function. Wataru et al. found that in patients with AF, the long non-coding RNA (lncRNA) FOXCUT strongly upregulates the expression of the transcription factor FOXC1. It exerts a post-transcriptional genetic regulation similar to that in carcinogenesis, prompting the transformation of endothelial cells into fibroblasts. This leads to atrial structural remodeling and the occurrence of arrhythmia (43). It has been pointed out (44) that FOXL1 can regulate a series of genes that enhance fibroblast function, including the transcriptional co-activator Yes -associated protein and/PDZ-binding motif (YAP/TAZ) marker genes and platelet- derived growth factor receptor-α. YAP/TAZ are downstream effectors of the Hippo signaling pathway and are crucial transcriptional co-activators. Some research reports that the FOX family is expressed in renal tubular epithelial cells during renal fibrosis and exhibits the ability to promote fibrosis and metabolic rewiring (45). The GATA2 transcription factor is a member of the GATA-binding transcription factor family and plays a key role in biological processes such as hematopoiesis and endocrine cell development, cell proliferation, and immune responses. For example, in terms of immune responses, GATA2 is involved in regulating the differentiation of dendritic cells, the proliferation of hematopoietic stem cells, the differentiation of IgE/mast cells, and the differentiation of granulocyte—monocyte progenitor cells (46, 47). Some studies have found that FOG2 is overexpressed in human heart failure. In addition, conditional overexpression of FOG2 in adult mouse cardiomyocytes leads to the occurrence of primary AF. As a protein that can bind directly to GATA2, FOG2 can cooperate with GATA2 to regulate the expression of target genes after they bind to each other (48). Studies have shown (49) that overexpression of GATA2 reduces the phagocytic ability of phagocytes and affects the maturation of phagosomes. A mouse model study found (50) that the expression of GATA2 in mice can enhance the production of inflammatory mediators by adipocytes, and in mouse macrophages, the toll-like receptor-4 (TLR4) signaling pathway up-regulates GATA2, and subsequently GATA2 cooperates with the mitogen-activated protein kinase (MAPK) signaling pathway to induce IL-1b transcription. Interestingly, YAP has been found to interact with the transcription factor GATA2, enhancing the transcription of aquaporin-2 (AQP2) in the renal collecting ducts, and controlling the final urine volume and osmotic pressure. When the YAP gene is deficient, the mRNA and protein abundance of Aqp2 in the kidneys is significantly reduced, leading to disorders in renal water—salt metabolism and further promoting the progression of kidney diseases (51). Some scholars have proposed that in kidney injury caused by ischemia-reperfusion injury, GATA2 positively regulates the expression of cytokines and chemokines in collecting duct cells, promoting disease progression. By inhibiting GATA2, the expression of inflammatory factors can be reduced, and kidney damage can be significantly alleviated (52).

Our study has discovered that PPBP, CXCL1, and RSAD2 promote the development of AF and CKD, among which the CXCLs/CXCR signaling pathway may play a crucial role. In addition, the related transcription factors FOXC1, FOXL1, and GATA2 also lead to disease progression by mediating the high expression of inflammatory factors, profibrotic factors, and immune cells. Based on the current research findings on the potential pathogenesis of these two diseases, these discoveries provide guiding suggestions for us to develop more effective treatment plans in clinical practice. Currently, in the standardized treatment of AF, the focus is mainly on three aspects: rhythm control, heart rate control, and anticoagulation therapy. Among them, pulmonary vein isolation (PVI) is a widely recognized treatment for rhythm control and serves as the cornerstone of catheter ablation for AF. Vinitha Nesapiragasan et al. (53) pointed out that even with persistent PVI, there is still a possibility of arrhythmia recurrence. For patients with ineffective PVI or recurrence after persistent PVI, catheter ablation strategies other than PVI can be adopted. Among these, renal denervation (RDN) can reduce the frequency of AF. In a study on a canine model by Wang et al. (54), the results showed that RDN inhibited the upward trend of TNF-α and IL-6 levels in the left atrium after prolonged right atrial pacing, and significantly alleviated the damage of myocardial fibrosis caused by chronic right atrial pacing. This demonstrated that catheter-based RDN could suppress the activity of the RAAS and atrial remodeling, including the significant increase in atrial fibrosis, inflammation, and apoptosis. In a randomized controlled study, it was found that among patients with paroxysmal AF and hypertension, compared with using catheter ablation alone, adding RDN significantly reduced the likelihood of AF recurrence (55). In addition, some studies have pointed out (56) that RDN inhibits the over-activation of the systemic sympathetic nervous system, providing strong protection against the occurrence and progression of kidney and heart diseases. For patients with both AF and CKD, in addition to conventional drug therapies, opting for RDN might be a favorable treatment option. But at present, RND mainly focuses on the treatment of hypertension (57). There are relatively few studies on using RND to treat AF and CKD. Larger-scale and more in-depth research needs to be carried out. However, the broad prospects of the clinical application of RND can be anticipated.

In summary, in the pathogenesis of AF and CKD, chronic inflammation can increase the risk of AF and CKD through promoting atrial remodeling, oxidative stress, fibrosis and other pathways. PPBP, CXCL1 and RSAD2 may play important roles in the occurrence and development of AF and CKD, among which the CXCLs/CXCR signaling pathway may play a crucial role. With a better understanding of the underlying mechanisms of AF and CKD, patients with AF-CKD will be better managed and treated.

### 4.1 Limitation

This study has certain limitations. First, due to the diverse sources, incompleteness and multi-sourced nature of biological data, during the data processing, the identification of biomarkers may be interfered by batch effects and missing data values. Second, in the analysis of gene sequencing data, phenomena such as gene horizontal transfer and mutation can lead to inaccurate results and over-fitting of the model, affecting the reliability of the model. Finally, due to the complexity of biological systems, the limitations of data analysis methods and the uncertainty of biological significance, false—positive or false—negative results may occur. Therefore, the specific functions and mechanisms may need to be further verified through experiments.

## 5 Conclusion

In this study, three biomarkers (PPBP, CXCL1, and RSAD2) were identified as diagnostic markers for AF-CKD patients. It was found that immune and inflammatory pathways accelerate the occurrence and development of AF-CKD, among which the CXCLs/CXCR signaling pathway may play a crucial role. FOXC1, FOXL1, and GATA2 may be potential therapeutic targets for AF combined with CKD. The correlations between PPBP, CXCL1, RSAD2, and immune cells may play important roles in the pathogenesis of AF-CKD. Whether these biomarkers can become new targets for molecular—targeted therapy in AF-CKD patients requires further research.

## Data Availability

The original contributions presented in the study are publicly available. These data can be found here: https://www.ncbi.nlm.nih.gov/geo/query/acc.cgi?acc, accession numbers: GSE79768, GSE37171, GSE120683, GSE14975.
